# Editorial: The role of circulating immune mediators in the crosstalk between cells of the immune system and cardiovascular system in CVDs

**DOI:** 10.3389/fimmu.2023.1197852

**Published:** 2023-04-11

**Authors:** Achille Anselmo, Veronica Bonalume, Angela Raucci

**Affiliations:** ^1^ Flow Cytometry Resource, Advanced Cytometry Technical Applications Laboratory (FRACTAL) Ospedale San Raffaele Scientific Institute, Milan, Italy; ^2^ Experimental Cardio-Oncology and Cardiovascular Aging Unit, Centro Cardiologico Monzino, Milan, Italy

**Keywords:** cardiovascular diseases, soluble molecules, immunity, immune mediators, cardiovascular system

Cardiovascular diseases (CVDs) remain the leading cause of morbidity and mortality in developed countries (1) and encompass multiple disorders including atherosclerosis, acute myocardial infarction (AMI) and stroke. Risk factors that drive CVD development include hyperlipidemia, arterial hypertension, diabetes mellitus, age, smoking, sedentary lifestyle, stress, or their combination. Mechanistically, chronic inflammation and immune cell activation play a fundamental role in the pathogenesis and progression of CVD. Indeed, strong evidence have demonstrated a key role of soluble molecules such as pro- and anti-inflammatory cytokines in the atherosclerotic plaque stability (2–4) in the myocardial dysfunctions (5, 6) and in the ischemic stroke outcome (7). Moreover, extracellular vesicles (EVs), released by several cell types, have been shown to regulate a multitude of functions in target cells, thus maintaining the cardiovascular balance or contributing to pathological changes in CVD (8–10).

In the Research Topic—”The Role of Circulating Immune Mediators in the Crosstalk Between Cells of the Immune System and Cardiovascular System in CVDs”, we provided a platform for exposing the main advancements in the ongoing CVD research. Six papers including both reviews and original articles are published in the volume.

Among the reviews, Li et al. described the role of the endothelial glycocalyx (eGCX) in lung diseases mainly focusing on the hepatopulmonary syndrome (HPS). The eGCX is a carbohydrate-rich layer lining the vascular endothelium. It is formed by soluble plasma components, bound together either directly or *via* soluble proteoglycans and/or glycosaminoglycans. The composition of eGCX is dynamic due to enzymatic degradation, *de novo* biosynthesis of new molecules and recruitments of soluble molecules from the bloodstream (11). Moreover, new evidence supports the role of the eGCX damage and fragmentation under inflammatory conditions in the pathogenesis of CVDs (12). Li et al. describe in detail the pathways activated by eGCX involved in HPS pathogenesis providing new insight into the present knowledge of this syndrome.

The second review focus on the association between psoriasis and CVD. Psoriasis is an autoimmune and inflammatory skin disorder that primarily affects the elbows, knees, scalp, umbilicus, and lower back, with sharply defined erythematous plaques covered in silvery-white scales (13). Beyond the skin, systemic inflammation might cause the insurgence of immune-mediated alterations eventually leading to important comorbidities including CV (14). Orlando et al. provided an overview of the putative circulating factors involved in the skin/endothelium/cardiovascular system crosstalk. Moreover, the manuscript offers a detailed description of the current knowledge on the circulating immune players contributing to the pathogenic link between psoriasis and CVD. Finally, the authors highlighted the importance of therapies with biologic drugs not only in controlling skin inflammation but also in the potential reduction of CV risk in psoriatic patients.

Among CVDs, atherosclerosis contributes to the majority of cardiovascular events (15). Nowadays, efforts to identify new diagnostic and prognostic biomarkers for arteriosclerotic cardiovascular disease (ASCVD) continue to grow with the aim of improving treatments and reducing healthcare costs. To this end, He et al. investigated the prognostic value of the systemic immune-inflammation index (SII) in a large cohort of ASCVD individuals. Of note, the SII combines the neutrophil/lymphocyte and platelet/lymphocyte ratios thus taking into account both immune and non-immune cells involved in the pathogenesis of atherosclerosis (16). Interestingly, the authors found a significant positive association of the baseline ln-SII (SII ln transform) and all-cause mortality supporting the notion of a key role of inflammation and the immune system in the initiation and resolution of atherosclerosis.

A collateral aspect of atherosclerosis-induced cardiovascular events is represented by the long-term prognosis following acute AMI. In this respect, Kalinskaya et al. addressed their studies on the role of inflammatory mediators beyond the pathogenesis and onset of infarction but focusing on their contribution to the consequent spontaneous reperfusion occurring in infarction-related artery. They investigated the role of 14 different circulating cytokines, correlated to chronic inflammation typically associate with atherosclerosis, to the probability of occlusion of the infarction-related artery in AMI patients. With the current paper, they proved a novel link between inflammatory response, functional state of endothelium, and clot formation.

Liao et al. contributed to the current volume describing how the circulating pro-inflammatory Interleukin-6 (IL-6) causes postoperative atrial fibrillation (POAF) *via* two parallel mechanisms. They previously reported that IL-6 predisposes to atrial fibrosis and fibrillation by inducing cardiac fibroblast activation (17). The current original article revealed the additional direct effect of IL-6 on calcium currents in cardiomyocytes. In particular, combining *in vitro* electrophysiology techniques and *ex vivo* optical mapping of Ca^++^ fluxes, they demonstrated that IL-6 mediates Ca^++^ handling abnormalities contributing to the development of POAF. Ca++ transient alternans, observed after IL-6 treatments can indeed represents the trigger for frequency-induced arrhythmia. This new mechanism appears to be more relevant compared to the atrial fibrosis induction and it might constitute a novel pharmacological approach for the prevention and treatment of POAF.

A new frontier of inflammation-correlated circulating factors is represented by the release of extracellular microvesicles (EMVs). EMVs could be characterized by the presence of tissue factor (TF) on their external surface that is able to guide them to specific target tissues. Arderiu et al. previously reported that activated endothelial cells (ECs) release TF enriched EMVs (TF-EMVs) that target monocytes and induce their differentiation into EC-like cells (18). With the current paper, the authors added to this scenario the intriguing role of microRNAs (miRNA) in this cross-talk paradigm. They observed that TF-EMVs released by ECs are characterized by an high content of miR-126, that in turn, regulates angiogenesis by targeting several components of the VEGF pathway, and downregulates inflammatory genes.

This Research Topic highlighted the complexity of the interaction between circulating inflammatory mediators and CVDs. Interestingly, the papers published in the current volume cover the study of this interaction in distinct pathological conditions that affect eventually the cardiovascular system. Even though the overall big picture leads to a common take home message that focus the attention on inflammatory mediators as putative prognostic and pathogenic co-factors, the extension to new direct and undirect mechanisms played by distinct cytokines point out to the necessity of further studies.

## Author contributions

AA, VB and AR wrote and edited the manuscript. All authors contributed to the article and approved the submitted version.
